# Sonoporation-Mediated Gene Transfection: A Novel Direction for Cell Reprogramming *In Vivo*

**DOI:** 10.3389/fbioe.2021.803055

**Published:** 2022-01-31

**Authors:** Meng Du, Yue Li, Zhiyi Chen

**Affiliations:** ^1^ The First Affiliated Hospital, Medical Imaging Centre, Hengyang Medical School, University of South China, Hengyang, China; ^2^ Institute of Medical Imaging, Hengyang Medical School, University of South China, Hengyang, China; ^3^ Laboratory of Ultrasound Molecular Imaging, The Third Affiliated Hospital of Guangzhou Medical University, Guangzhou, China

**Keywords:** ultrasound, cell reprogramming, gene delivery, regenerative medicine, physical stimulation

## Introduction

Cell reprogramming is of great significance to biomedical science, especially in regenerative medicine application. The classical mode called indirect reprogramming is to obtain stem cells or induced pluripotent stem cells (iPSCs) and stimulate them to become mature differentiated cells, which has been applied in cardiac disease, neuron disease, etc. For example, with transfection of four transcriptional factors Oct4, Sox2, Klf4, and c-Myc (Yamanaka factors), somatic cells could be reprogrammed into iPSCs and further be stimulated to differentiate into mature functional cells (Takahashi and Yamanaka, 2006; Takahashi et al., 2007).

In contrast with indirect reprogramming, direct reprogramming has received lots of attention in recent years, due to its unique advantage of fast, efficient, and low risk of tumorigenesis. Direct reprogramming refers to the application of transcription factor overexpression, noncoding RNA delivery, or small molecule delivery to promote the direct transformation of cells into target differentiated cells. In other words, compared with indirect reprogramming, direct reprogramming does not have to go through the stage of pluripotent stem cells or progenitor cells, which means it is easier to perform during cell differentiation. Lately, direct reprogramming has been applied as a therapeutic strategy to many diseases. For example, in ophthalmology, with application of five small molecules, Mahato et al. successfully reprogrammed fibroblasts into rod photoreceptor-like cells *in vitro*, which were further transplanted to restore vision of blind mice (Mahato et al., 2020). In another study, direct reprogramming was applied to wound healing *in vivo*. Kurita et al. (2018) proved that with the introduction of a combination of four epidermal growth-related factors into the wound site, cells inside the wound were reprogrammed into epidermal cells, which enabled non-invasive wound healing and the function of new born skin was the same as the normal skin.

The greatest value of direct reprogramming is that it makes cell reprograming *in vivo* possible to perform (Sekiryu and Matsuda, 2021). The common method for *in vivo* reprograming rely on virus-based strategy, which transfect transcriptional factor gene into targeted cells. The most common used vector for gene transfection could be classified as viral and nonviral vectors (Chong et al., 2021). Viral vector has the advantage of high efficiency of gene transfection, while exogenous genes may be inserted randomly into the host cell genome, which threatens the integrity and safety of the cell genome (Geis et al., 2017). Nonviral vectors, such as liposome and nanoparticle, could reduce the rate of host immunogenicity response, which has received more concerns recently. Wang et al. developed a novel nonviral delivery system for cardiac reprograming, which consists of nanoparticles and biofilm. In detail, to enhance the targeting efficiency of this system, mesoporous silicon nanoparticles were decorated with neutrophil-mimicking membranes, which was modified with folic acid peptide. With high efficiency delivery of miRNA1, 133, 208, and 499 *in vivo*, cardiac fibroblasts in myocardial infarction area could be reprogramed into induced cardiomyocyte-like cells, and cardiac function of injury area finally recovers (Wang et al., 2021). However, compared with viral vectors, relatively low transfection efficiency limited the application of the nonviral vector.

Sonoporation is a special type of nonviral physical transfection strategy. Compared with other physical transfection modes, such as electroporation and gene microinjection, sonoporation has shown great application potential for gene transfection *in vivo*, due to the advantage of high specificity, deep penetration, and low cost (Tachibana et al., 1999). With gas-filled microbubbles (MBs) as cavitation nuclei and gene vector, ultrasound, able to trigger MBs rapidly oscillating and disrupt cell membrane to form transient pores (sonoporation), then enhances cellular membrane permeability and promotes gene entering into cytoplasm (Helfield et al., 2016; Rong et al., 2018; Meng et al., 2019). Sonoporation was considered as a non-invasive and nonviral approach to deliver functional molecules, such as pDNA, siRNA, or molecular drug, to overcome the physiological or pathological barriers for the purpose of preventing or treating diseases (Delalande et al., 2015; Bez et al., 2018; Tran et al., 2019).

With sonoporation, gene in acoustic responsive vector, such as MBs, could be release quickly and enter into targeted tissue or cells. Based on this theory, sonoporation-based direct reprogramming strategy was applied in diabetes treatment. In this research, Yang et al. applied SonoVue microbubble, which is a kind of commercial contrast agent used in clinics, as vector for hydrodynamic gene Pdx1, Neurog3, and MafA. First of all, in order to optimize the transfection rate, related parameters, including acoustic pressure, pulse repetition frequency, and plasmid concentration were tested and the acoustic parameter was set at 1 MPa acoustic pressure, 20 cycle pulses, and the irradiation duration was set at 5 min with an interval of 6 s. With the ultrasound irradiation at the liver after the gene-loaded MBs administration, it was shown that the hepatocytes have been reprogrammed into insulin-producing cells in a diabetic model successfully. After treatment, the glucose levels decreased significantly while the insulin levels were enhanced, which indicated that diabetic symptoms were alleviated (Yang et al., 2020). Accordingly, sonoporation was successfully applied in direct cell reprograming *in vivo*, and provide a non-invasive, safe, and efficient reprograming mode for regenerative medicine.

Nowadays, with the development of bioengineering science, ultrasound could not only be applied as a diagnosis method for clinics but also get more attention in its application in biological function regulation, substance triggered release, ablation, and so on. The continuous maturation of sonoporation technology also promotes its application in clinics (Carpentier et al., 2016; Dimcevski et al., 2016; Sitta and Howard, 2021). However, as for cell reprogramming applications, sonoporation still has its limitation. First of all is the choice of gene vector. During the process of sonoporation, vector is not only used to carry genes, but also play the role as cavitation nucleus to improve permeability of tissue or cells during ultrasound irradiation. Application of commercial MBs, such as SonoVue, is a potential solution. In many clinical trials involving sonoporation-mediated drug delivery, MBs are administered separately from chemotherapy drugs. However, it is not appropriate for gene transfection since genes are easily degraded during circulation. Although a lot of novel vectors with high gene loading were developed, the biocompatibility of these chemically synthesized vectors is still a problem to be solved. Research on inducing cell reprogramming by facilitating small molecules into target tissues through sonoporation may be an interesting topic. Second is the standardization of sonoporation operation mode. Sonoporation transfection involves many key parameters, including acoustic pressure, frequency, irradiation time, and so on. The optimal parameters reported by different researches vary greatly, which may be caused by the various ultrasound devices applied. For example, the optimal parameters used in the above research (1 MPa acoustic pressure, 20 cycle pulses, 5 min for irradiation duration, and interval of 6 s) are quite different from those used in our previous research (Yu et al., 2018; Li et al., 2021) (0.6 MPa, 30% duty cycle, and 5 min for irradiation duration; 2.0 W/cm^2^, 50% duty cycle, and 1 min for irradiation duration). Therefore, the operation mode of sonoporation is difficult to be unified and requires individual customization. Last but not the least, the transfection efficiency. Cell reprograming, especially *in vivo* cell reprograming, has high requirements on gene transfection efficiency. There is still a big gap in transfection rate between virus strategy and sonoporation. Combining sonoporation with other transfection strategies, such as virus transfection and chemical transfection, is a potential solution. In addition, in order to optimize transfection efficiency, it is valuable to explore the influence of biological effects of ultrasound on mitochondria and cell fate, and reveal its biological mechanism. In other words, although sonoporation transfection still faces many challenges, based on the integration and collaboration of various disciplines (Huang, 2020), there is no doubt that sonoporation will open a new chapter for regenerative medicine in the clinic.
